# Supplementary material for: Resolving the full spectrum of human genome variation using Linked-Reads
Source: Genome Res. 2019 Apr;29(4):635–45. doi: 10.1101/gr.234443.118 (PMC6442396; doi:10.1101/gr.234443.118)
Supplement: Supplemental Material [file supp_gr.234443.118_Supplemental_longranger-2.2.zip › longranger-2.2.tar.bz2!/lr2.2-public/lib/stan/cmdstan/stan/doc/api/html/classstan_1_1math_1_1seq__view_3_01_t_00_01std_1_1vector_3_01_t_01_4_01_4.html]

Stan: stan::math::seq\_view< T, std::vector< T > > Class Template Reference


|  |  |
| --- | --- |
|  | Stan  2.5.0  probability, sampling & optimization |


- Main Page
- Related Pages
- Namespaces
- Classes
- Files

- Class List
- Class Index
- Class Hierarchy
- Class Members

All Classes Namespaces Files Functions Variables Typedefs Enumerations Enumerator Friends Macros Pages

- stan
- math
- seq\_view< T, std::vector< T > >

Public Member Functions |
List of all members

stan::math::seq\_view< T, std::vector< T > > Class Template Reference

`#include <seq_view.hpp>`

|  |  |
| --- | --- |
| Public Member Functions | |
|  | seq\_view (typename pass\_type< std::vector< T > >::type x) |
|  | |
| pass\_type< T >::type | operator[] (int n) const |
|  | |
| int | size () const |
|  | |

## Detailed Description

### template<typename T> class stan::math::seq\_view< T, std::vector< T > >

Definition at line 137 of file seq\_view.hpp.

## Constructor & Destructor Documentation

template<typename T >

|  |  |  |  |  |  |
| --- | --- | --- | --- | --- | --- |
| stan::math::seq\_view< T, std::vector< T > >::seq\_view | ( | typename pass\_type< std::vector< T > >::type | *x* | ) |  |

inline

Definition at line 141 of file seq\_view.hpp.

## Member Function Documentation

template<typename T >

|  |  |  |  |  |  |
| --- | --- | --- | --- | --- | --- |
| pass\_type<T>::type stan::math::seq\_view< T, std::vector< T > >::operator[] | ( | int | *n* | ) | const |

inline

Definition at line 145 of file seq\_view.hpp.

template<typename T >

|  |  |  |  |  |
| --- | --- | --- | --- | --- |
| int stan::math::seq\_view< T, std::vector< T > >::size | ( |  | ) | const |

inline

Definition at line 148 of file seq\_view.hpp.

---

The documentation for this class was generated from the following file:

- src/stan/math/seq\_view.hpp


---

[ Stan Home Page ]

*© 2011–2014,
Stan Development Team.*
